# Flowering Newsletter 2022

**DOI:** 10.1093/jxb/erac269

**Published:** 2022-08-11

**Authors:** Rainer Melzer

**Affiliations:** Flowering Newsletter Editor, JXB School of Biology and Environmental Science and Earth Institute, University College Dublin, Ireland

**Keywords:** Angiosperms, Arabidopsis, Asteraceae, epigenetics, flower development, jasmonate, monocots, orchids

**I’m writing this editorial while drinking coffee. I have just had a slice of bread for breakfast. Later, I may eat an apple during a break. Coffee beans, wheat grains, apples: the list could go on. Fruits—and by extension flowers—have a relevance in our lives that can hardly be underestimated. Beyond providing calories, fruits and flowers are also beautiful. Many flowers evolved to attract pollinators, but almost everyone enjoys the sight of a field of flowers.**

The analysis of plant reproduction can be easily justified by the economic and societal importance of crops and ornamental plants. In addition, climate change threatens many ecosystems around the world, and, with plants at the heart of many of those ecosystem plant research projects and particularly research on plant reproduction, flower development and flowering time are more important than ever.

Research on flowering plants is thus geared towards addressing some of the most pressing issues of our time; yet, for me and I suppose for many other researchers, flowers also have an inherent fascination that drives our interest in analysing them. Flowers are beautiful, complex, modular, morphologically diverse reproductive structures. We do not know how exactly they originated during evolution, how floral diversity was established, or what the detailed gene regulatory circuits are that govern flower development. However, our understanding is increasing, and this year’s Flowering Newsletter is a tour de force through some of the most significant research advances in floral biology.

One of the most pertinent questions relates to the age of flowering plants. Numerous quite diverging age estimates have been published in the past, and Sauqet *et al*. (2022) provide detailed insight as to why we may have to live with some uncertainty for the foreseeable future. For now, the authors suggest we settle on an age estimate of 140–270 million years ago for the origin of the crown angiosperms.

As outlined by Sauquet *et al.* (2022), the age of the angiosperms has far-reaching implications for other highly important questions, such as the co-evolution of plants with animals and the diversification of non-flowering plant groups, but of course the diversification of angiosperms has also attracted so much interest because flowering plants represent by far the most species-rich group of land plants, dominating almost all terrestrial ecosystems (Benton *et al.*, 2022). Among flowering plants, two families stand out as being particularly species rich: the Orchidaceae (orchids) and the Asteraceae (sunflower family) (Bánki *et al.*, 2022). Among the most prominent morphological novelties of Asteraceae is the inflorescence, which can be composed of hundreds of small flowers (termed florets) organized in a spiral pattern forming the head or capitulum (Zhao *et al.*, 2016). This intriguing arrangement of flowers has fascinated researchers for a long time, and recently computational models and experimental data led to a better understanding of the patterning processes in the Asteraceae head (Zhang *et al.*, 2021). In the 2022 Flowering Newsletter issue, Prusinkiewicz *et al.* (2022) provide an overview over this patterning process, and also explain what happens when head formation goes awry: while Asteraceae heads are usually circular, so-called fasciated heads that are elliptical or ribbon-like are occasionally observed (Prusinkiewicz *et al.*, 2022). The authors outline how patterning of fasciated heads can be modelled with surprising accuracy and thus open up this problem to further biological inquiry.

The plant hormone auxin plays a critical role in the patterning of the Asteraceae head, but of course many hormones contribute to flowering time regulation and flower development. Among those, jasmonate is a relatively unexplored player, and Zhao *et al.* (2022) provide a detailed and convincing account of how this hormone plays an important role in the control of flowering time.

While organization of flowers in a head is a remarkable morphological innovation in the Asteraceae, orchids stand out because of their diversity in floral organ morphology. Most orchid flowers are zygomorphic with highly specialized petaloid organs. Li *et al.* (2022) review how genomics and molecular genetics have greatly increased our understanding of orchid reproductive morphology. A large number of genes controlling orchid flower development were recently identified and functionally characterized. It is now increasingly clear that core pathways of flower development are conserved across large evolutionary distances, but that at the same time gene duplications and subfunctionalizations have contributed to the morphological diversification of the orchid perianth (Li *et al.*, 2022).

The petaloid organs of orchids are spectacular, but other angiosperms also possess quite elaborate petals. Fu *et al.* (2022) dissect the developmental process through which petals arise. They show that four main paths of modification can explain the development of elaborate petals (Fu *et al.*, 2022). Similar to Prusinkiewicz *et al.* (2022), this work shows that a combination of modelling and experimental data can be a very powerful approach to dissect patterning processes.

Asteracea and Orchidacea are the largest plant families, but Poaceae (grasses) can claim the first place for feeding the world. The three main staple crops—maize, rice, and wheat—are all grasses (FAO, 2022). The cereals are so important because of the endosperm of the grain, which is extremely rich in starch (Huang *et al.*, 2021). The grain, of course, is the fruit of the cereals, developing from the fertilized carpel. Surprisingly, despite its outstanding importance, the evolutionary history of the grass gynoecium (from which the grain develops) is not entirely clear (Sokoloff *et al.*, 2022). Sokoloff *et al.* (2022) present evidence that the grass gynoecium consists of three highly integrated carpels, two of which are sterile.

Of course, though a number of reviews in the Flowering Newsletter 2022 issue are dedicated to a diversity of topics and species, Arabidopsis research is alive and well (Juárez-Corona and de Folter, 2021; Krizek *et al.*, 2021). Beth Krizek describes her excitement about her research on the regulation of floral size in Arabidopsis (Krizek, 2022), and Nobutoshi Yamaguchi reviews epigenetic processes involved in flower development (Yamaguchi, 2022). These articles highlight how incredibly detailed our understanding of molecular genetic processes in Arabidopsis is, and that much more remains to be learned.

What becomes clear from the 2022 issue of the Flowering Newsletter is that our understanding of flower development and evolution continues to improve at a fast pace and is increasingly moving beyond traditional model plants such as Arabidopsis or rice. The knowledge the community has generated and continues to generate on traditional model plants is extremely useful to decipher gene regulatory circuits and developmental processes in other species, because gene functions are often at least partially conserved. Next-generation sequencing and CRISPR have massively facilitated this process (Preston, 2020), and now enable detailed insights into flower development and evolution, as exemplified also by articles in the Flowering Newsletter issue.

However, as powerful as this approach is, it also can be limiting in terms of identifying novel genes and gene regulatory circuits. Forward genetic approaches are in many plants still impeded by, for example, long generation times or low seed yields. For species not limited by these considerations, genetic mapping of natural allelic variants or ethyl methanesulfonate (EMS)-induced mutations can be a promising way forward (Liu *et al.*, 2021; Trubanová *et al.*, 2022). Modelling and detailed comparative morphological studies can be absolutely crucial to deliver new insights, too. For example, as pointed out by Sokoloff *et al.* (2022), the complicated evolutionary history of the grass gynoecium would have been impossible to infer without comparative studies.

The papers in the Flowering Newsletter issue thus show the enormous progress that research on flowers is making, but also outline how much more remains to be learned. Many more coffees will be consumed in the process—and plenty of fun while making new discoveries is guaranteed.
